# Effects of once-daily oral orforglipron on weight and metabolic markers: a systematic review and meta-analysis of randomized controlled trials

**DOI:** 10.20945/2359-4292-2023-0469

**Published:** 2024-09-11

**Authors:** Carine Lütkemeyer, Eric Pasqualotto, Rafael Oliva Morgado Ferreira, Matheus Pedrotti Chavez, Ilmar Petris, Henrique Vilar dos Santos, Julia Murbach Wille, Alexandre Hohl, Marcelo Fernando Ronsoni, Simone van de Sande-Lee

**Affiliations:** 1 Pesquisador Independente Florianópolis SC Brasil Pesquisador Independente, Florianópolis, SC, Brasil; 2 Divisão de Medicina Universidade Federal de Santa Catarina Florianópolis SC Brasil Divisão de Medicina, Universidade Federal de Santa Catarina, Florianópolis, SC, Brasil; 3 Divisão de Medicina Universidade Federal do Paraná Curitiba PR Brasil Divisão de Medicina, Universidade Federal do Paraná, Curitiba, PR, Brasil

**Keywords:** Orforglipron, body weight, obesity, metabolism

## Abstract

The aim of this study is to assess the effects of once-daily oral orforglipron on weight and metabolic markers in adult patients. PubMed, Embase, Cochrane Library, and ClinicalTrials.gov databases were systematically searched until February 2024 for randomized controlled trials (RCTs) comparing orforglipron versus placebo or other anti-obesity medications in adult patients. Weighted mean differences (WMDs) for continuous outcomes and risk ratios (RRs) or risk differences for binary endpoints were computed, with 95% confidence intervals (CIs). Heterogeneity and risk of bias were assessed with I^2^ statistics and Rob-2, respectively. Statistical analyses were performed using R, version 4.2.2. A total of four studies were included, comprising 815 patients, of whom 620 (76.1%) were prescribed orforglipron. Compared with placebo, orforglipron reduced body weight (WMD -6.14 kg, 95% CI -9.62 to -2.66 kg), body mass index (WMD -2.87 kg/m^2^, 95% CI -4.65 to -1.10 kg/m^2^), and waist circumference (WMD -5.32 cm, 95% CI -9.13 to -1.51 cm). More patients treated with orforglipron than placebo achieved a weight loss of ≥ 5% (RR 3.31, 95% CI 2.23-4.93), ≥ 10% (RR 5.24, 95% CI 2.07-13.31), and ≥ 15% (RR 9.53, 95% CI 1.26-71.89). The most common adverse events were related to the gastrointestinal tract. In this meta-analysis, the use of once-daily oral orforglipron by adult patients was associated with a significant decrease in body weight, as compared with placebo, with an increase in non-severe gastrointestinal adverse events. Phase 3 RCTs are expected to shed further light on the efficacy and safety of once-daily oral orforglipron over the long term.

## INTRODUCTION

Obesity is a chronic disease associated with reduced quality of life and increased morbidity and mortality (1-3). It is estimated that approximately 42% of the world’s population will live with overweight or obesity by the year 2025 (4). Recently, glucagon-like peptide-1 receptor agonists (GLP-1 RAs), a class of antidiabetic and anti-obesity medications, have gained growing emphasis in the management of obesity and overweight, as well as coexisting weight-related conditions (5-7). These drugs are peptide-based and are administered via subcutaneous injections or orally (8). However, subcutaneous administration presents challenges in use and treatment maintenance (9). In addition to the possible discomfort during administration, there are difficulties in producing the drug and its device, which has led to a global shortage in recent years due to a large increase in demand (10). Semaglutide, the only orally administered drug available, uses an absorption enhancer and must be taken on an empty stomach with no more than 120 mL of water, leaving a 30-minute interval before ingesting food, drinks, and other medications, which may impair treatment adherence (11).

Orforglipron, an oral non-peptide GLP-1 RA, represents a new generation of GLP-1 RAs that are easier to produce and can be administered orally without food restriction (7,11). By reducing appetite and delaying gastric emptying, this drug has the potential to induce weight loss and could become an interesting alternative to injectable GLP-1 RAs in the treatment of obesity and overweight (7).

A few phase 2 randomized controlled trials (RCTs) have recently been published comparing once-daily oral orforglipron with placebo treatment in adult patients (7,11). Herein, we performed the first comprehensive systematic review and meta-analysis evaluating the safety and efficacy of orforglipron as an anti-obesity medication.

## METHODS

This systematic review followed the Preferred Reporting Items for Systematic Reviews and Meta-Analysis (PRISMA) guidelines (12). The study protocol was registered in the International Prospective Register of Systematic Reviews (PROSPERO) with registration number CRD42023458940 (13).

### Eligibility criteria

Studies with the following criteria were included: (A) RCTs, (B) comparing once-daily oral orforglipron with placebo or other anti-obesity medications, (C) involving adult patients (aged ≥ 18 years), and (D) reporting at least one of the outcomes of interest. Studies with the following criteria were excluded: (A) non-RCTs, (B) overlapping populations, and (3) RCTs with ongoing recruitment or without published results.

### Search strategy

PubMed, Embase, Cochrane Library, and ClinicalTrials.gov were systematically searched from inception to February 15, 2024, with the following search terms: orforglipron OR LY3502970. Abstracts of major endocrinology meetings from the past 3 years were searched for eligible studies. Aiming for the inclusion of additional studies, references of the included articles and systematic reviews were evaluated.

### Data extraction

Two authors (C.L. and M.P.C.) independently extracted baseline characteristics and data outcomes following predefined search criteria. Disagreements were resolved by consensus between three authors (C.L., M.P.C., and S.S.L.).

### Endpoints

The outcomes of interest were body weight (kg); weight reduction (%); body mass index (BMI; kg/m^2^); weight reduction of ≥5%, ≥10%, and ≥15%; waist circumference (cm); total cholesterol (%); triglycerides (%); low-density lipoprotein (LDL) cholesterol (%); high-density lipoprotein (HDL) cholesterol (%); alanine transaminase (ALT; %); aspartate aminotransferase (AST; %); pulse rate (bpm); fasting serum glucose (mg/dL); glycated hemoglobin (HbA1c; %); systolic blood pressure (mmHg); diastolic blood pressure (mmHg); alkaline phosphatase (%); serious adverse events; diarrhea; nausea; vomiting; constipation; dyspepsia; and cardiac disorders.

### Risk of bias

The Cochrane Collaboration tool for assessing risk of bias in randomized trials (Rob-2) was used to assess individual RCTs (14). Each trial received a risk of bias score – high, low, or some concerns – in five domains: randomization process, deviations from the intended interventions, missing outcomes, measurement of the outcome, and selection of reported results. Two independent authors conducted the risk of bias assessment (C.L. and E.P.), and disagreements were resolved unanimously with the senior author (S.S.L.).

### Statistical analysis

The treatment effects for continuous outcomes were compared using weighted mean differences (WMDs), and binary endpoints were evaluated using risk ratios (RRs) or risk differences (RDs) along with 95% confidence intervals (CIs). Heterogeneity was assessed with the Cochran Q-test and I^2^statistics; p values < 0.10 and I^2^ values > 25% were considered indicative of significant heterogeneity (15). DerSimonian and Laird random-effects models were used for all endpoints (16). For data handling and conversion, the guidelines of the Cochrane Handbook for Systematic Reviews of Interventions were used (17). Statistical analyses were performed using the R software, version 4.2.2 (R Core Team, 2021, Vienna, Austria).

### Sensitivity analysis

Leave-one-out procedures were used to identify influential studies and their effects on the pooled estimates, evaluating the heterogeneity. This procedure was carried out by removing data from one study and reanalyzing the remaining data. When pooled effect size p values changed from significant to nonsignificant, or vice versa, study dominance was assigned.

### Quality assessment

The quality of evidence was assessed according to the Grading of Recommendations, Assessment, Development, and Evaluation (GRADE) guidelines (18,19). Very-low-quality, low-quality, moderate-quality, or high-quality evidence grades were designed for the outcomes based on the risk of bias, inconsistency of results, imprecision, publication bias, and magnitude of treatment effects.

## RESULTS

### Study selection and characteristics

**Figure 1:**
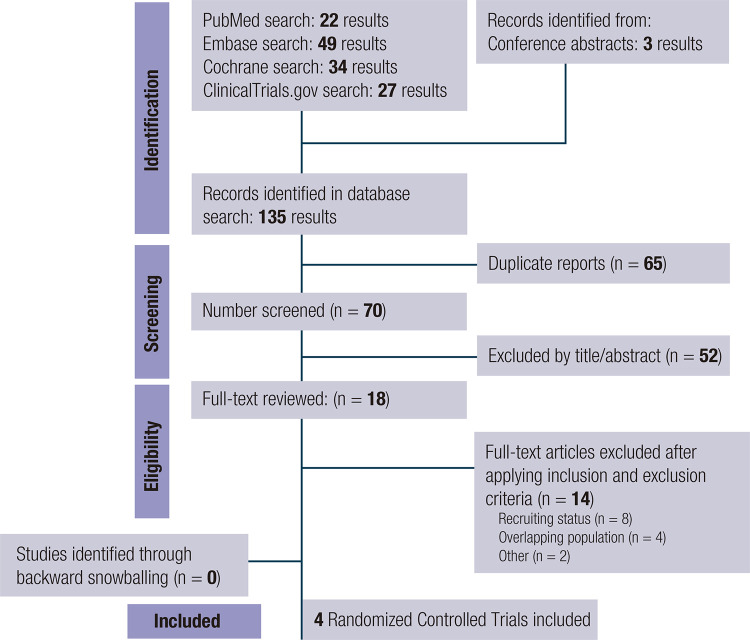
Preferred Reporting Items for Systematic Reviews and Meta-Analysis (PRISMA) flow diagram of study screening and selection. The search strategy in PubMed, Embase, Cochrane, and ClinicalTrials.gov yielded 135 studies, of which 18 were fully reviewed for inclusion and exclusion criteria. A total of four studies were included in the meta-analysis.

As illustrated in Figure 1, the search strategy yielded 135 results. After removing duplicates and ineligible studies by title or abstract, 18 studies were fully reviewed for inclusion and exclusion criteria. Of these, four were included in this meta-analysis (7,8,11,20). A total of 815 patients were included, of whom 620 (76.1%) were treated with orforglipron, 50 (6.1%) with dulaglutide, and 145 (17.8%) with placebo. Their mean age ranged from 54.0 to 59.0 years, and their mean body weight ranged from 84.0 to 108.9 kg. Table 1 details the baseline characteristics of the studies included in the meta-analysis.

**Table 1:** Baseline characteristics of the studies included in the meta-analysis

Study	Trial phase	Follow-up (weeks)	OG doses (mg)	Sample sizes OG/CG	Female OG/CG (%)	Age OG/CG, (years), mean (SD)	Body weight OG/CG (kg), mean (SD)	BMI OG/CG (kg/m^2^), mean (SD)	Waist circumference OG/CG (cm), mean (SD)	HbA1c OG/CG (%), mean (SD)	Fasting serum glucose OG/CG (mg/dL), mean (SD)	Type 2 diabetes OG/CG (%)
Frias 2023	Phase 2	26	3; 12; 24; 36; 45	278/55/50♯	110 (39.6)/ 27 (49)/ 20 (40)♯	59.0 (9.2)/ 58.3 (9.5)/ 58.8 (10.2)♯	100.3 (21.9)/ 102 (18.8)/ 98.8 (22.1)♯	35.0 (6.9)/ 35.8 (6.2)/ 35.4 (8)♯	113.6 (15.0)/ 115 (12.4)/ 114 (16.4)♯	8.1 (0.8)/ 8.1 (0.9)/ 8 (0.7)♯	166.1 (38.4)/ 172 (42.9)/ 167.6 (38)♯	278 (100)/ 55 (100)/ 50 (100)♯
Pratt 2023	Phase 1a	4	0.3/ 1/ 3/ 6‡; 2/ 4/ 6†; 2/ 4/ 8/ 16†; 2/ 5/ 12/ 24†	69/23	29 (31.5)¶	42.8¶	84.0¶	28.6¶	N/A	<6.5§	82.7 (3.4)/ N/A	N/A
Pratt 2023	Phase 1b	12	3/ 6/ 9†; 3/ 6/ 12/ 15†; 3/ 6/ 12/ 21†; 3/ 6/ 12/ 21/ 27†; 3/ 6/ 9/ 21/ 36/ 45†	51/17	19 (37.2)/ 7 (41.2)	58.5 (6.3)/ 56.0 (6.0)	88.4 (15.06)/ 90.29 (20.04)	30.89 (4.09)/ 31.31 (4.86)	N/A	8.03 (0.91)/ 8.09 (0.75)	N/A	51 (100)/ 17 (100)
Wharton 2023	Phase 2	36	12; 24; 36; 45	222/50	132 (59.4)/ 29 (58.0)	54.2 (11.0)/ 54.0 (8.8)	108.9 (26.2)/ 107.6 (25.2)	37.9 (7.0) 37.8 (6.5)	117.2 (16.2)/ 115.5 (15.4)	5.6 (0.4)/ 5.6 (0.4)	96 (11.3)/ 97.2 (10.2)	N/A

♯The data are presented as orforglipron group/placebo group/dulaglutide group. §Inclusion criteria were patients with HbA1c level < 6.5%. ¶Data indicating the total number of patients, not classified between control and intervention. ‡Single ascending doses (SADs). †Multiple ascending doses (MADs). Abbreviations: BMI, body mass index; CG, control group; HbA1c, glycated hemoglobin; N/A, not available; OG, orforglipron group; SD, standard deviation.

### Pooled analysis of all studies

#### Weight and metabolic markers outcomes

**Figure 2:**
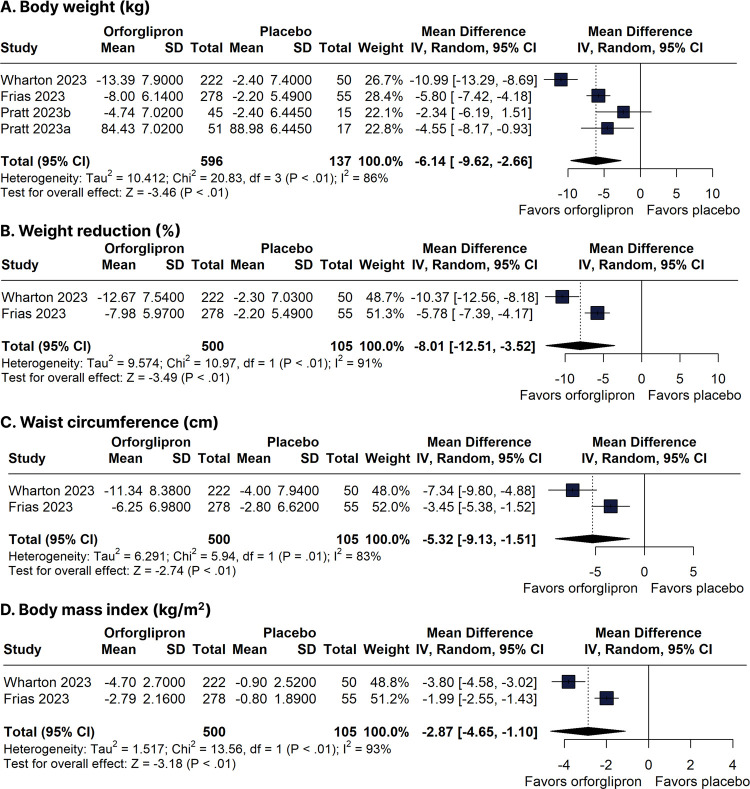
Forest plots of pooled comparisons between orforglipron and placebo. (A) Body weight (kg). (B) Weight reduction (%). (C) Waist circumference (cm). (D) Body mass index (kg/m2).

Body weight reduction was greater in the orforglipron group compared with the placebo group (WMD -6.14 kg, 95% CI -9.62 to -2.66 kg; four trials, 733 participants, low certainty of evidence) (Figure 2A). In a pooled analysis including only phase 2 RCTs, the reduction in body weight was also greater in the orforglipron group (WMD -8.33 kg, 95% CI -13.41 to -3.24 kg; two trials, 605 participants) compared with the placebo group. The percentage weight reduction was greater with orforglipron (WMD -8.01%, 95% CI -12.51 to -3.52%; two trials, 605 participants) than with placebo (Figure 2B). Patients treated with orforglipron (*versus* placebo) also had greater reductions in waist circumference (WMD -5.32 cm, 95% CI -9.13 to -1.51 cm; two trials, 605 participants, evidence of very low certainty) and BMI (WMD -2.87 kg/m^2^, 95% CI -4.65 to -1.10 kg/m^2^; two trials, 605 participants, evidence of very low certainty) (Figures 2C and 2D).

**Figure 3:**
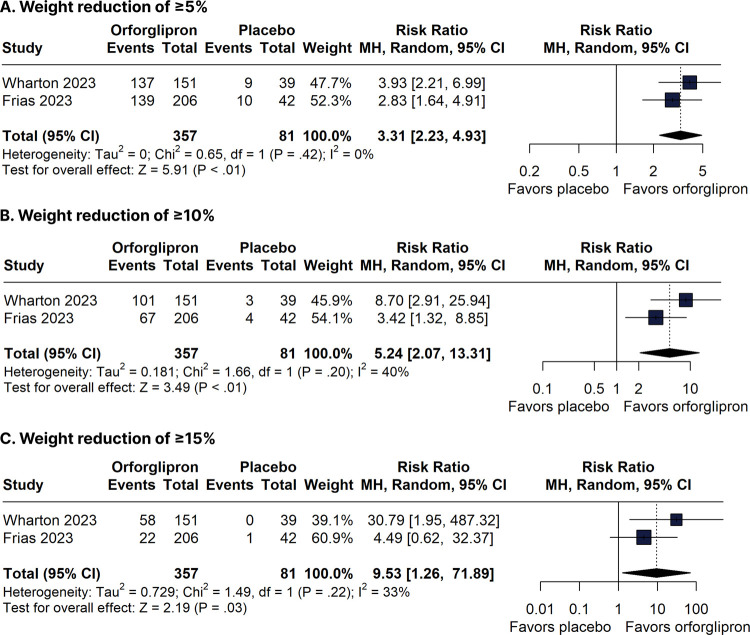
Forest plots of pooled comparisons between orforglipron and placebo with risk ratios. (A) Weight reduction of ≥ 5%. (B) Weight reduction of ≥ 10%. (C) Weight reduction of ≥ 15%.

In the statistical analysis using RRs, the orforglipron compared with the placebo group had a greater number of patients with weight loss of ≥ 5% (RR 3.31, 95% CI 2.23-4.93; two trials, 438 participants, evidence of very low certainty), ≥ 10% (RR 5.24, 95% CI 2.07-13.31; two trials, 438 participants, evidence of very low certainty), and ≥ 15% (RR 9.53, 95% CI 1.26-71.89; two trials, 438 participants, evidence of very low certainty) (Figures 3). In the statistical analysis using RDs, the orforglipron group compared with the placebo group also had a greater number of patients with weight loss of ≥ 5% (RD 0.56, 95% CI 0.32-0.79; two trials, 438 participants) and ≥ 10% (RD 0.41, 95% CI 0.06-0.77; two trials, 438 participants) (Supplementary Figures 1A and 1B). However, the analysis showed a neutral effect between groups in the number of patients who achieved a weight loss of ≥ 15% (RD 0.23, 95% CI -0.07-0.54; two trials, 438 participants) (Supplementary Figure 1C).

Reductions in total cholesterol (WMD -7.34%, 95% CI -13.14 to -2.55%; two trials, 605 participants), triglycerides (WMD -12.88%, 95% CI -20.46 to -5.30%; two trials; 605 participants), LDL cholesterol (WMD -9.01%, 95% CI -15.30 to -2.72%; two trials, 605 participants), and ALT (WMD -9.29%, 95% CI -15.80 to -2.78%; two trials, 605 participants) levels favored orforglipron over placebo (Supplementary Figures 2A-D). However, orforglipron was associated with an increased pulse rate (WMD 8.78 bpm, 95% CI 6.16 to 11.41 bpm; four trials, 733 participants) (Supplementary Figure 3A).

Orforglipron (*versus* placebo) reduced fasting serum glucose (WMD -23.16 mg/dL, 95% CI -44.09 to -2.23 mg/dL; three trials, 461 participants), HbA1c (WMD -0.84%, 95% CI 1.54 to -0.14%; three trials, 673 participants, evidence of very low certainty), and systolic blood pressure (WMD -3.14 mmHg, 95% CI -6.17 to -0.12 mmHg; four trials, 731 participants) (Supplementary Figures 3B-3D). The analysis showed a neutral effect between groups regarding diastolic blood pressure (WMD 0.56 mmHg, 95% CI -1.26 to 2.38 mmHg; four trials, 731 participants), alkaline phosphatase (WMD -0.05%, 95% CI -3.56 to 3.47%; two trials, 605 participants), HDL cholesterol (WMD 1.09%, 95% CI -2.50 to 4.68%; two trials, 605 participants), and AST (WMD -3.70%, 95% CI -9.19 to 1.79%; two trials, 605 participants) levels (Supplementary Figures 4A-4D).

Only one of the analyzed studies compared orforglipron versus dulaglutide 1.5 mg (11). In the orforglipron group, weight reductions of ≥ 5%, ≥ 10%, and ≥ 15% were achieved by 67.5%, 32.5%, and 10.7% of the patients, respectively, while in the dulaglutide group, the corresponding percentages were 34.9%, 4.7%, and 2.3%, respectively (11).

Compared with dulaglutide 1.5 mg, the orforglipron doses of 12, 24, 36, and 45 mg were superior in reducing HbA1c and fasting serum glucose levels, body weight, and BMI values. However, the results indicated a neutral effect between orforglipron 3 mg and dulaglutide 1.5 mg for these outcomes. Additionally, orforglipron 24, 36, and 45 mg significantly reduced waist circumference compared with dulaglutide 1.5 mg, but the results showed a neutral effect between orforglipron 3 and 12 mg and dulaglutide 1.5 mg (11).

#### Adverse events

Compared with placebo, orforglipron increased the rates of nausea (RR 5.34, 95% CI 2.83-10.08; three trials, 673 participants), vomiting (RR 5.97, 95% CI 2.84-12.58; three trials, 673 participants), and constipation (RR 4.52, 95% CI 1.71-11.98; two trials, 605 participants) (Supplementary Figures 5A-5C). However, the results showed neutral effects between groups regarding serious adverse events (RR 0.77, 95% CI 0.20-2.99; three trials, 673 participants), diarrhea (RR 1.69, 95% CI 0.72-3.96; three trials, 673 participants), dyspepsia (RR 1.83, 95% CI 0.80-4.18; three trials, 673 participants), and cardiac disorders (RR 2.56, 95% CI 0.80-8.26; three trials, 639 participants) (Supplementary Figures 5D and 6A-6C).

## Sensitivity analysis

Most outcomes did not show stability in their results, with changes in statistical significance when each individual study was removed and the effect estimates were reanalyzed. However, in outcomes related to body weight reduction, results were stable, without major changes in significance with the removal of each individual study. The changes observed may be due to the low number of RCTs for each outcome.

## Risk of bias and quality of evidence

Supplementary Figure 7 outlines the individual appraisal of each RCT included in this systematic review and meta-analysis. Overall, all studies were deemed to have a moderate risk of bias (7,8,11,20).

According to the GRADE assessment, one outcome evaluated was classified as low-quality evidence: body weight. A very-low-quality evidence was assigned for the outcomes of BMI, weight reduction of ≥ 5%, weight reduction of ≥ 10%, weight reduction of ≥ 15%, waist circumference, and HbA1c. The main domains responsible for reducing the quality of evidence of the outcomes were risk of bias, inconsistency of results due to heterogeneity, and imprecision due to the small number of RCTs included in the statistical analysis. Quality assessment is detailed in Supplementary Material 2.

## DISCUSSION

In this systematic review and meta-analysis of four RCTs involving 815 patients, we assessed the efficacy and safety of once-daily oral orforglipron as an anti-obesity medication, compared with placebo or other anti-obesity medications. Our key findings were as follows: (A) orforglipron reduced body weight, BMI, and waist circumference; (B) orforglipron increased the proportion of patients achieving weight loss of ≥ 5%, ≥ 10%, and ≥ 15% in the statistical analysis using RRs, while in the analysis using RDs, orforglipron (*versus* placebo) had a greater proportion of patients achieving weight loss of ≥ 5% and ≥ 10%; (C) orforglipron reduced fasting serum glucose and HbA1c levels; and (D) orforglipron did not increase severe adverse events. However, the very-low-quality to low-quality evidence of the results must be considered, in addition to differences in the patients’ baseline characteristics and the small number of included studies.

Increasingly, GLP-1 RAs are being incorporated into obesity or overweight treatment alongside lifestyle changes (5). Recently introduced as an anti-obesity medication, semaglutide 2.4 mg demonstrated a placebo-adjusted weight reduction of 12.4%, with almost one-third of individuals achieving a weight loss of 20% or more (21,22). Additionally, tirzepatide, a glucose-dependent insulinotropic polypeptide (GIP)/GLP-1 dual agonist, has proven efficacy in weight reduction and was recently approved by the Food and Drug Administration (FDA) for treating type 2 diabetes (T^2^D) and obesity (5,23,24). The SURMOUNT-1 trial, including individuals with obesity without diabetes, showed that weekly tirzepatide at doses of 5 mg, 10 mg, and 15 mg led to an average weight loss of 15%, 19%, and 21%, respectively, compared with 3% in the placebo group at 72 weeks (22).

Clinical benefits of weight reduction require a sustained loss of 3%-5% of body weight, with additional impacts when higher percentage losses are achieved (21,22,25). Bariatric surgery is currently the therapeutic option that leads to more substantial and lasting weight reduction in patients with obesity. Recently, comparable weight losses have been achieved in studies of anti-obesity drugs (26,27). In our meta-analysis, orforglipron was associated with an increase in the proportion of patients achieving a clinically relevant weight reduction. Similar results were reported in previous meta-analyses with weekly subcutaneous semaglutide and once-daily oral semaglutide (21,28). However, it should be noted that oral semaglutide must be taken on an empty stomach, without any food, liquid, or other medication for at least 30 minutes after ingestion, posing a challenge to its proper use. In contrast, orforglipron, lacking a peptide in its chemical formulation, does not require such precautions to enhance absorption and may be a potential alternative in obesity treatment (7,11,29). Of note, direct and indirect statistical comparisons between orforglipron and semaglutide have not been performed yet.

In a previous meta-analysis evaluating the effect of GLP-1 RAs in individuals with obesity without T2D, weekly administration of subcutaneous semaglutide led to a 12.4 kg reduction in body weight, whereas liraglutide, another GLP-1 RA, resulted in a 5.3 kg weight reduction (30). Therefore, our findings suggest that daily orforglipron, with an average weight reduction of 6.14 kg (or 8.33 kg, when excluding phase 1 studies) compared with placebo, demonstrates a concordant effect with other GLP-1 RAs, without the inconvenience of subcutaneous administration. Of note, the phase 1 and 2 RCTs included in this meta-analysis had short follow-ups, and the weight curves indicated that a plateau in weight loss was not reached, suggesting that greater percentages of weight loss may be observed in studies with longer follow-ups. Furthermore, it is important to consider that our meta-analysis included individuals with T2D, a population in which the effect of anti-obesity medications is typically smaller than in patients without T2D (31).

Besides the observed reductions in body weight, GLP-1 RAs also positively impact individuals’ quality of life by improving metabolic parameters (21,30). Our findings showed a favorable effect of orforglipron on lipid profile compared with placebo. Reductions in total cholesterol, triglycerides, and LDL cholesterol decrease the occurrence of cardiovascular events (32). However, orforglipron caused an increase in baseline heart rate, previously described with other GLP-1 analogs (6,33). Although GLP-1 RAs have been documented to lead to an increase in heart rate, this effect has not been associated with increased cardiovascular or arrhythmia risk (34). Regarding glycemic control, orforglipron reduced fasting serum glucose and HbA1c levels when compared with placebo, although not all patients included in our analysis had T2D. The studies by Frias and cols. and Pratt and cols., which exclusively evaluated patients with T2D, demonstrated a reduction in HbA1c levels with orforglipron compared with placebo (11,20). Notably, in the study by Frias and cols., orforglipron was superior to dulaglutide in reducing fasting serum glucose and HbA1c levels (11).

In patients with T2D, GLP-1 RAs have proven cardiovascular benefits (35-37). The recent SELECT trial demonstrated, for the first time, a reduction in the composite outcome of cardiovascular mortality, nonfatal myocardial infarction, or nonfatal stroke with weekly subcutaneous semaglutide in individuals with overweight or obesity and cardiovascular disease, but without diabetes (38). Furthermore, tirzepatide significantly reduced major adverse cardiovascular events and cardiovascular death compared with placebo in a pooled analysis of the SURMOUNT-1 and SURPASS trials (39). The effects of other GLP-1 RAs on cardiovascular outcomes remain to be evaluated in patients with obesity or overweight. However, the results of the SELECT trial highlight the importance of treating obesity to reduce cardiovascular risk (38).

A previous network meta-analysis evaluating approved drugs for overweight and obesity treatment revealed that GLP-1 analogs (semaglutide and liraglutide) might cause adverse effects leading to treatment discontinuation (27). However, drugs with a higher risk of adverse events leading to discontinuation were phentermine-topiramate and naltrexone-bupropion (27). Furthermore, daily semaglutide and liraglutide, in contrast to their weekly regimens, had higher withdrawal rates due to adverse events when these drugs were compared with placebo (6). In our study, a higher rate of gastrointestinal adverse events was encountered in patients treated with orforglipron. Nonetheless, it was reassuring that severe adverse events were not significantly increased with orforglipron.

This study has some limitations. First, the analysis was based on a limited number of phase 1 and 2 RCTs and different orforglipron doses, which may have influenced the effect size found in our results. Although we found many studies meeting the inclusion criteria, some had ongoing recruitment. Also, four conference abstracts included populations that overlapped with those of the studies in the present meta-analysis. Second, there was moderate to high heterogeneity in some of the outcomes analyzed. However, we performed a leave-one-out sensitivity analysis to assess the stability of our results. Overall, the results were mostly unstable, mainly due to the small number of studies that had reported the outcomes of interest. Third, the RCTs evaluated in this meta-analysis presented different inclusion criteria, which may have influenced our results, especially since not all individuals had obesity. Fourth, only one study compared orforglipron with other anti-obesity drugs, which prevented a more thorough analysis. Fifth, due to a lack of evidence, we were unable to assess the cost-utility of orforglipron. Finally, although this study represents the largest pooled analysis of patients treated with orforglipron to date, it was underpowered to establish its metabolic, cardiovascular, and clinical effects.

In conclusion, this meta-analysis found that the use of once-daily oral orforglipron led to a higher weight loss than placebo in adult patients, with an increase in nonsevere gastrointestinal adverse events. Furthermore, orforglipron showed a reduction in fasting serum glucose and HbA1c levels when compared with placebo. However, the very-low-quality to low-quality evidence of the results and limitations of this study must be considered. Phase 3 RCTs are expected to shed further light on the efficacy and safety of once-daily oral orforglipron over the long term.
