# Supplementary material for: Effects of once-daily oral orforglipron on weight and metabolic markers: a systematic review and meta-analysis of randomized controlled trials
Source: Arch Endocrinol Metab. 2024 Sep 11;68:e230469. doi: 10.20945/2359-4292-2023-0469 (PMC11460968; doi:10.20945/2359-4292-2023-0469)
Supplement: Supplementary file 1 [file 2359-4292-aem-68-e230469-suppl01.pdf]

SUPPLEMENTARY MATERIALS

Supplementary Material 1

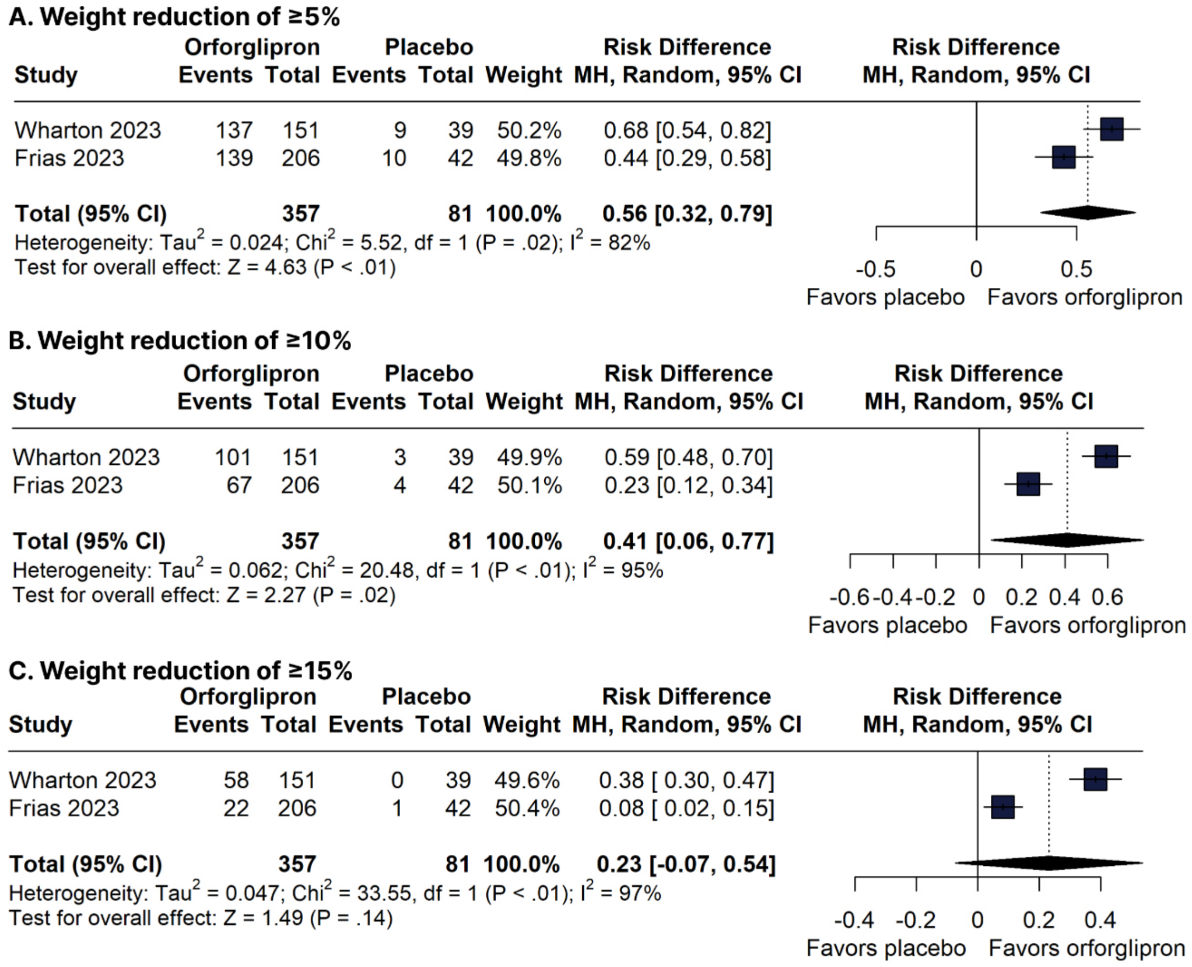

Supplementary Figure 1. Forest plots of pooled comparisons between orforglipron and placebo, with risk differences. (**A**) Weight reduction of ≥ 5%. (**B**) Weight reduction of ≥ 10%. (**C**) Weight reduction of ≥ 15%.

Copyright© AE&M all rights reserved.

A. Total cholesterol

Study	Orforglipron Mean	Orforglipron SD	Orforglipron Total	Placebo Mean	Placebo SD	Placebo Total	Weight	Mean Difference IV, Random, 95% CI
Wharton 2023	-7.87	16.1700	222	-2.50	16.2600	50	54.4%	-5.37 [-10.35, -0.39]
Frias 2023	-5.19	18.5600	278	5.60	20.6900	55	45.6%	-10.79 [-16.68, -4.90]
**Total (95% CI)**			**500**			**105**	**100.0%**	**-7.84 [-13.14, -2.55]**

Heterogeneity: $Tau^2 = 6.944$; $Chi^2 = 1.90$, df = 1 (P = .17); $I^2 = 47\%$
Test for overall effect: Z = -2.91 (P < .01)

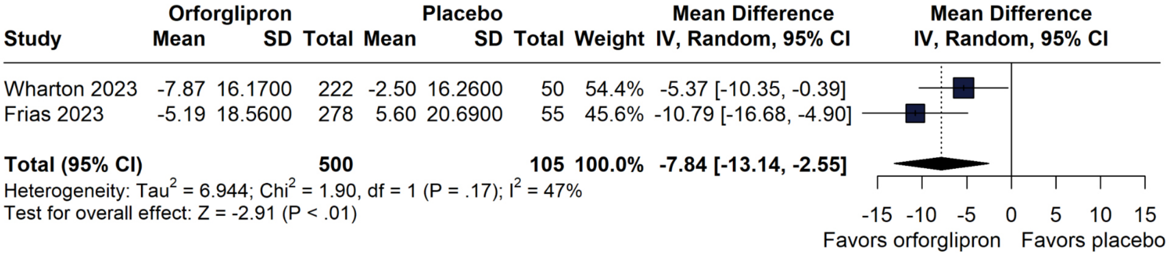

B. Triglycerides

Study	Orforglipron Mean	Orforglipron SD	Orforglipron Total	Placebo Mean	Placebo SD	Placebo Total	Weight	Mean Difference IV, Random, 95% CI
Wharton 2023	-11.13	32.6900	222	0.80	34.6500	50	51.8%	-11.93 [-22.45, -1.41]
Frias 2023	-9.30	33.7400	278	4.60	38.4900	55	48.2%	-13.90 [-24.82, -2.98]
**Total (95% CI)**			**500**			**105**	**100.0%**	**-12.88 [-20.46, -5.30]**

Heterogeneity: $Tau^2 = 0$; $Chi^2 = 0.06$, df = 1 (P = .80); $I^2 = 0\%$
Test for overall effect: Z = -3.33 (P < .01)

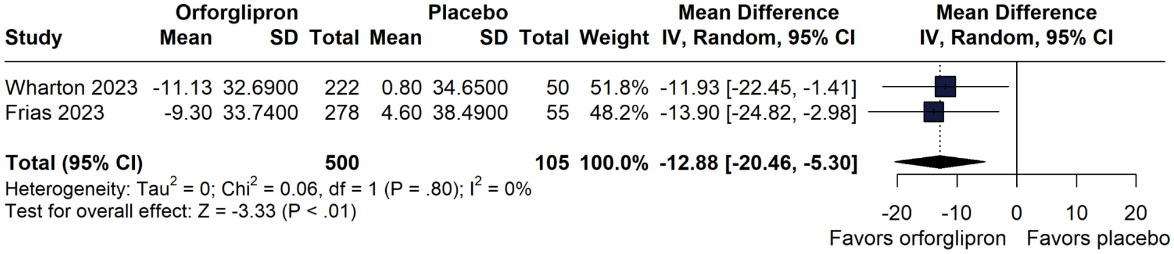

C. Low-density lipoprotein (LDL)

Study	Orforglipron Mean	Orforglipron SD	Orforglipron Total	Placebo Mean	Placebo SD	Placebo Total	Weight	Mean Difference IV, Random, 95% CI
Wharton 2023	-9.63	24.8600	222	-2.90	25.4600	50	65.4%	-6.73 [-14.51, 1.05]
Frias 2023	-7.83	32.9000	278	5.50	37.7500	55	34.6%	-13.33 [-24.03, -2.63]
**Total (95% CI)**			**500**			**105**	**100.0%**	**-9.01 [-15.30, -2.72]**

Heterogeneity: $Tau^2 = 0$; $Chi^2 = 0.96$, df = 1 (P = .33); $I^2 = 0\%$
Test for overall effect: Z = -2.81 (P < .01)

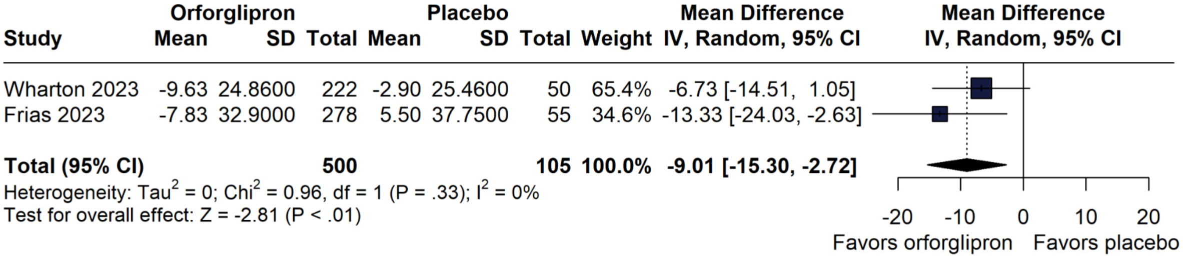

D. Alanine transaminase (ALT)

Study	Orforglipron Mean	Orforglipron SD	Orforglipron Total	Placebo Mean	Placebo SD	Placebo Total	Weight	Mean Difference IV, Random, 95% CI
Wharton 2023	-24.08	24.7200	222	-14.20	28.2800	50	58.8%	-9.88 [-18.37, -1.39]
Frias 2023	-20.55	32.1100	278	-12.10	35.6000	55	41.2%	-8.45 [-18.59, 1.69]
**Total (95% CI)**			**500**			**105**	**100.0%**	**-9.29 [-15.80, -2.78]**

Heterogeneity: $Tau^2 = 0$; $Chi^2 = 0.04$, df = 1 (P = .83); $I^2 = 0\%$
Test for overall effect: Z = -2.80 (P < .01)

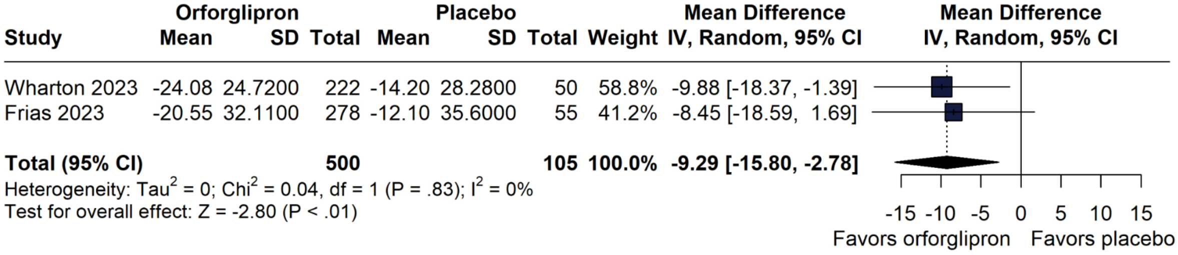

Supplementary Figure 2. Forest plots of pooled comparisons between orforglipron and placebo. (**A**) Total cholesterol. (**B**) Triglycerides. (**C**) Low-density lipoprotein (LDL) cholesterol. (**D**) Alanine transaminase (ALT).

Copyright© AE&M all rights reserved.

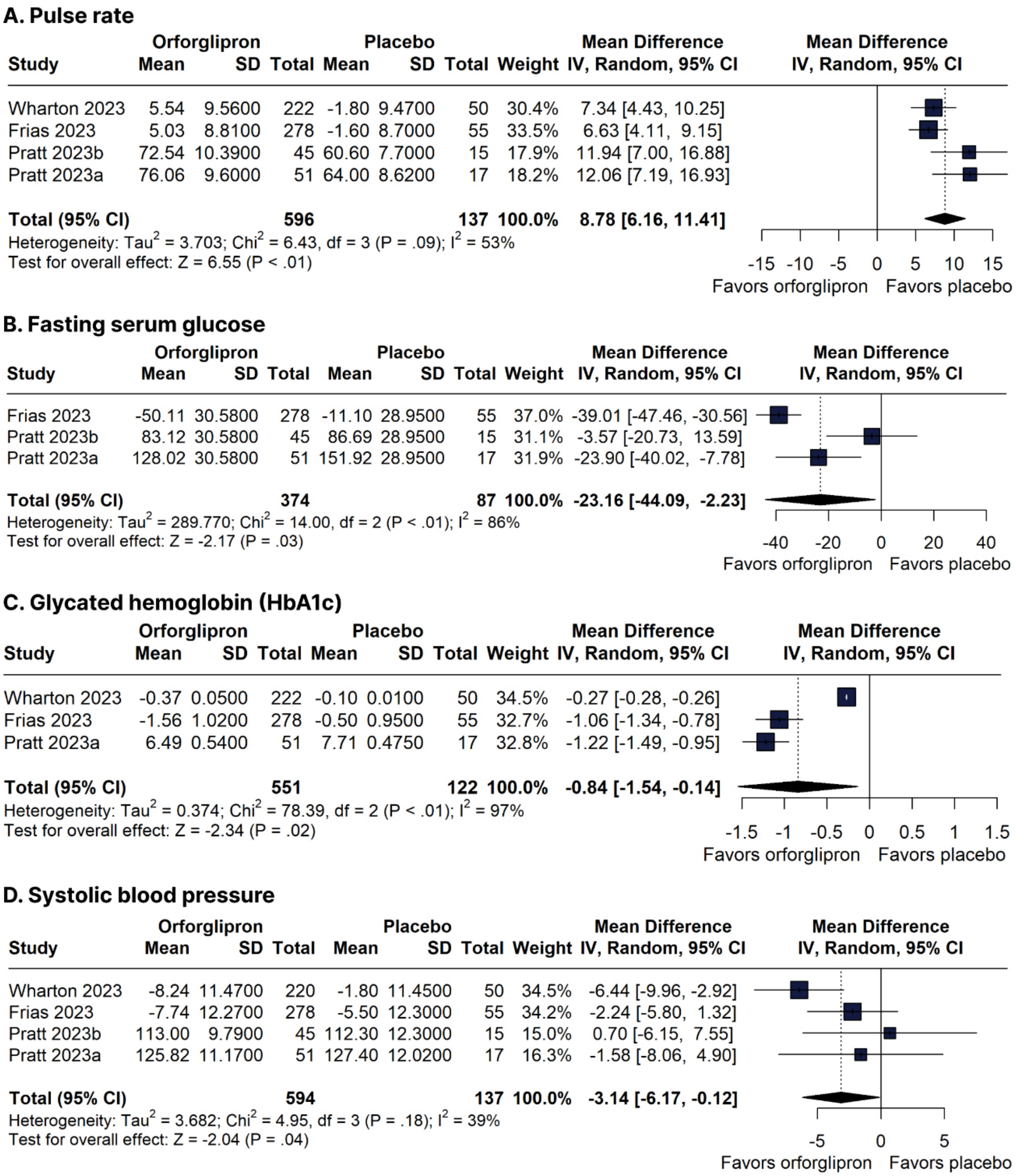

Supplementary Figure 3. Forest plots of pooled comparisons between orforglipron and placebo. (**A**) Pulse rate. (**B**) Fasting serum glucose. (**C**) Glycated hemoglobin (HbA1c). (**D**) Systolic blood pressure.

Copyright© AE&M all rights reserved.

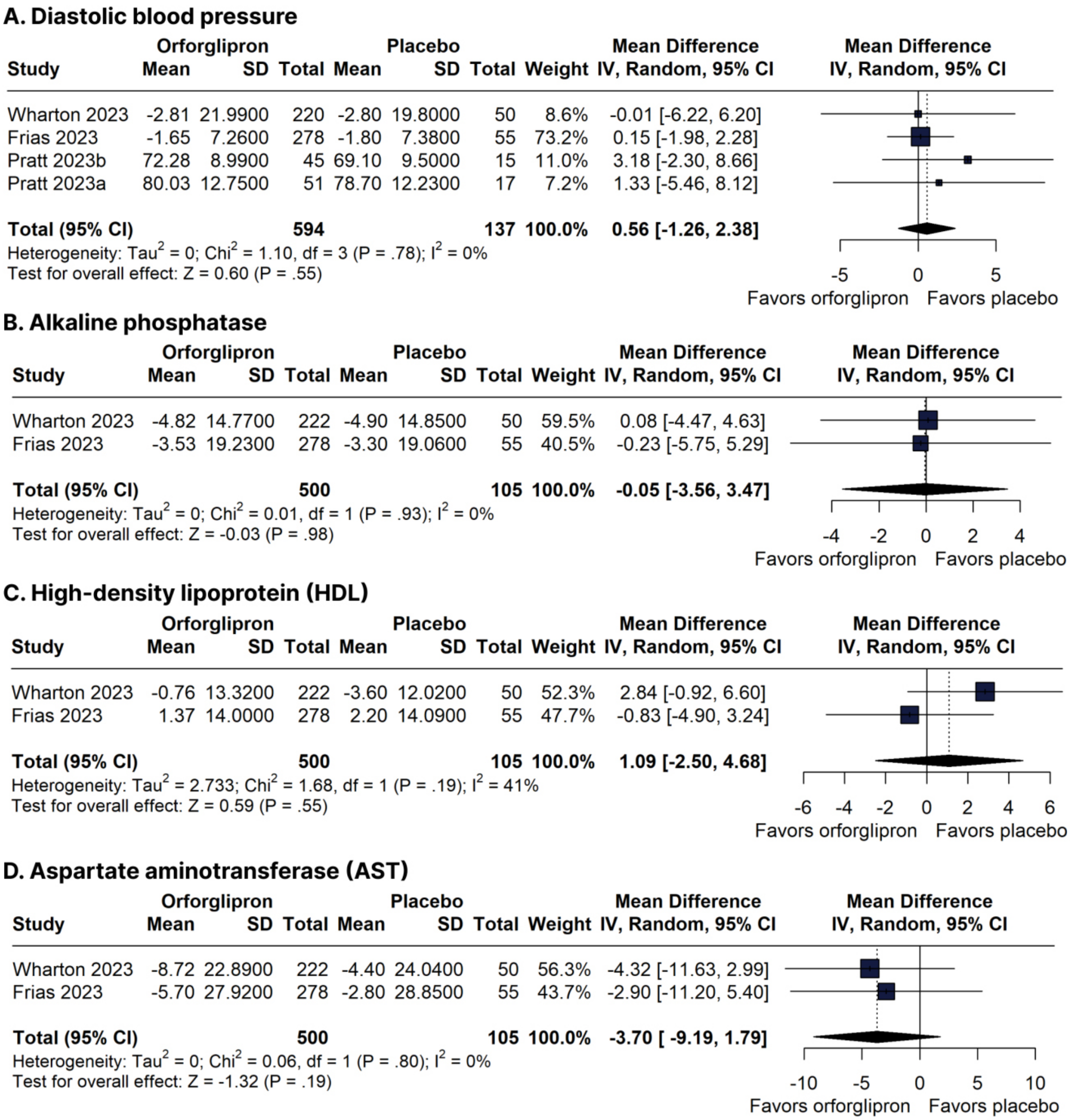

Supplementary Figure 4. Forest plots of pooled comparisons between orforglipron and placebo. (**A**) Diastolic blood pressure. (**B**) Alkaline phosphatase. (**C**) High-density lipoprotein (HDL) cholesterol. (**D**) Aspartate aminotransferase (AST).

Copyright© AE&M all rights reserved.

A. Nausea

Study	Orforglipron Events	Orforglipron Total	Placebo Events	Placebo Total	Weight	Risk Ratio MH, Random, 95% CI
Wharton 2023	106	222	5	50	56.7%	4.77 [2.06, 11.09]
Frias 2023	85	278	3	55	32.4%	5.61 [1.84, 17.09]
Pratt 2023a	25	51	1	17	10.9%	8.33 [1.22, 56.95]
**Total (95% CI)**		**551**		**122**	**100.0%**	**5.34 [2.83, 10.08]**

Heterogeneity: $Tau^2 = 0$; $Chi^2 = 0.28$, df = 2 (P = .87); $I^2 = 0\%$
Test for overall effect: Z = 5.18 (P < .01)

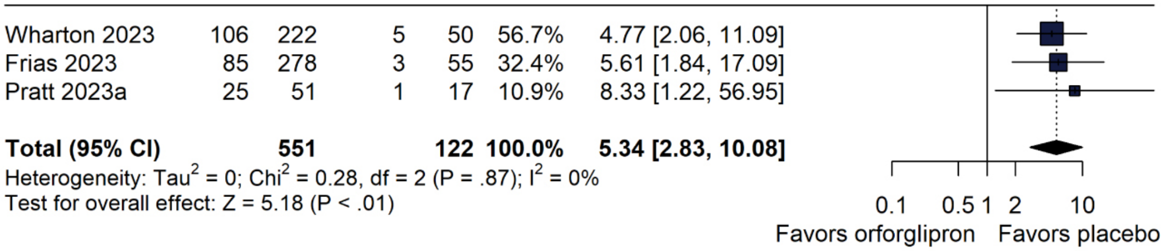

B. Vomiting

Study	Orforglipron Events	Orforglipron Total	Placebo Events	Placebo Total	Weight	Risk Ratio MH, Random, 95% CI
Wharton 2023	106	222	5	50	78.1%	4.77 [2.06, 11.09]
Frias 2023	62	278	1	55	14.5%	12.27 [1.74, 86.60]
Pratt 2023a	22	51	0	17	7.3%	15.58 [1.00, 243.84]
**Total (95% CI)**		**551**		**122**	**100.0%**	**5.97 [2.84, 12.58]**

Heterogeneity: $Tau^2 = 0$; $Chi^2 = 1.35$, df = 2 (P = .51); $I^2 = 0\%$
Test for overall effect: Z = 4.70 (P < .01)

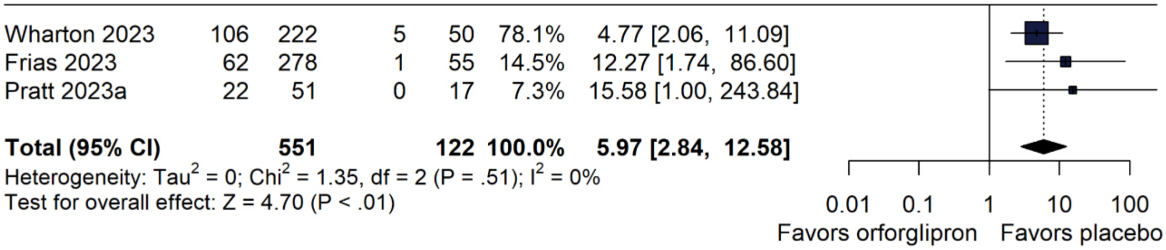

C. Constipation

Study	Orforglipron Events	Orforglipron Total	Placebo Events	Placebo Total	Weight	Risk Ratio MH, Random, 95% CI
Wharton 2023	54	222	3	50	75.5%	4.05 [1.32, 12.44]
Frias 2023	32	278	1	55	24.5%	6.33 [0.88, 45.36]
**Total (95% CI)**		**500**		**105**	**100.0%**	**4.52 [1.71, 11.98]**

Heterogeneity: $Tau^2 = 0$; $Chi^2 = 0.15$, df = 1 (P = .70); $I^2 = 0\%$
Test for overall effect: Z = 3.03 (P < .01)

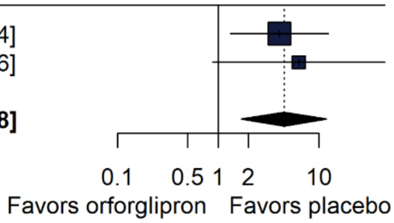

D. Serious adverse events

Study	Orforglipron Events	Orforglipron Total	Placebo Events	Placebo Total	Weight	Risk Ratio MH, Random, 95% CI
Wharton 2023	7	222	0	50	19.5%	3.43 [0.20, 59.10]
Frias 2023	12	278	3	55	64.2%	0.79 [0.23, 2.71]
Pratt 2023a	0	51	1	17	16.3%	0.12 [0.00, 2.71]
**Total (95% CI)**		**551**		**122**	**100.0%**	**0.77 [0.20, 2.99]**

Heterogeneity: $Tau^2 = 0.353$; $Chi^2 = 2.49$, df = 2 (P = .29); $I^2 = 20\%$
Test for overall effect: Z = -0.38 (P = .71)

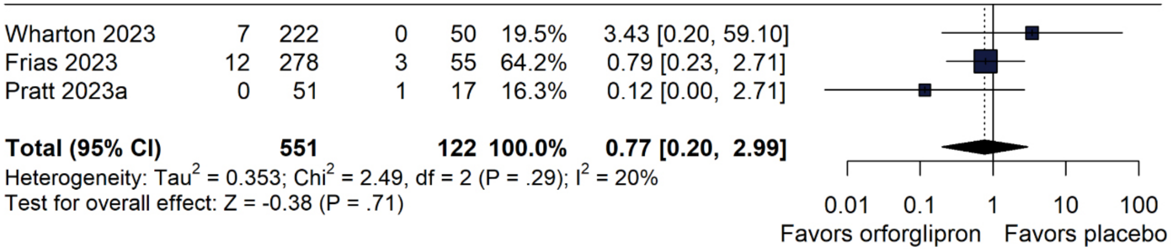

Supplementary Figure 5. Forest plots of pooled comparisons between orforglipron and placebo. (**A**) Nausea. (**B**) Vomiting. (**C**) Constipation. (**D**) Serious adverse events.

Copyright© AE&M all rights reserved.

A. Diarrhea

Study	Orforglipron Events	Orforglipron Total	Placebo Events	Placebo Total	Weight	Risk Ratio MH, Random, 95% CI
Wharton 2023	51	222	5	50	39.2%	2.30 [0.97, 5.46]
Frias 2023	54	278	4	55	35.4%	2.67 [1.01, 7.07]
Pratt 2023a	5	51	3	17	25.5%	0.56 [0.15, 2.08]
**Total (95% CI)**		**551**		**122**	**100.0%**	**1.69 [0.72, 3.96]**

Heterogeneity: $Tau^2 = 0.287$; $Chi^2 = 4.07$, df = 2 (P = .13); $I^2 = 51\%$
Test for overall effect: Z = 1.21 (P = .23)

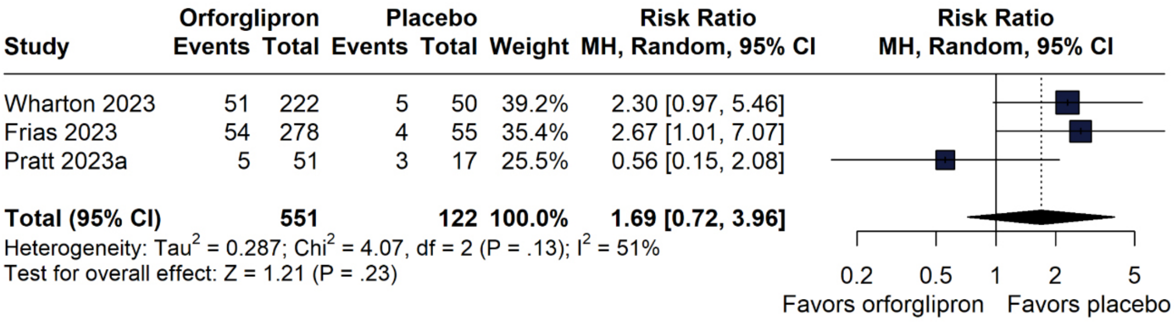

B. Dyspepsia

Study	Orforglipron Events	Orforglipron Total	Placebo Events	Placebo Total	Weight	Risk Ratio MH, Random, 95% CI
Wharton 2023	19	222	3	50	49.1%	1.43 [0.44, 4.63]
Frias 2023	22	278	2	55	33.9%	2.18 [0.53, 8.99]
Pratt 2023a	8	51	1	17	17.0%	2.67 [0.36, 19.80]
**Total (95% CI)**		**551**		**122**	**100.0%**	**1.83 [0.80, 4.18]**

Heterogeneity: $Tau^2 = 0$; $Chi^2 = 0.37$, df = 2 (P = .83); $I^2 = 0\%$
Test for overall effect: Z = 1.43 (P = .15)

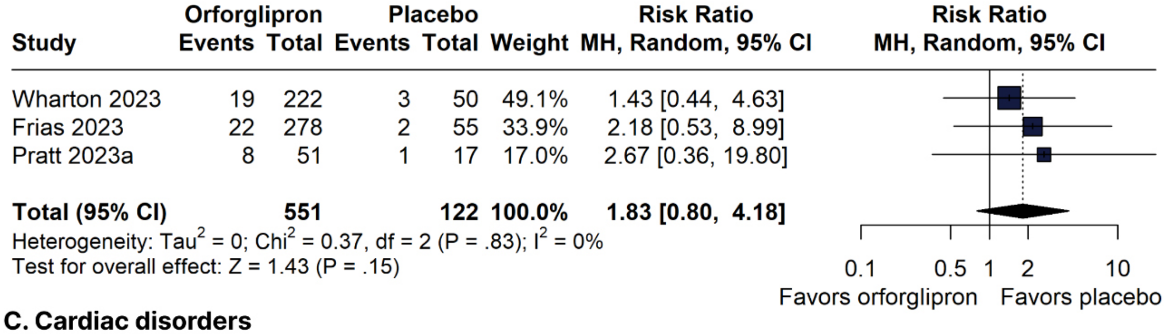

C. Cardiac disorders

Study	Orforglipron Events	Orforglipron Total	Placebo Events	Placebo Total	Weight	Risk Ratio MH, Random, 95% CI
Wharton 2023	19	222	0	50	16.9%	8.92 [0.55, 145.30]
Frias 2023	23	212	3	55	83.1%	1.99 [0.62, 6.38]
**Total (95% CI)**		**434**		**105**	**100.0%**	**2.56 [0.80, 8.26]**

Heterogeneity: $Tau^2 = 0.075$; $Chi^2 = 1.06$, df = 1 (P = .30); $I^2 = 6\%$
Test for overall effect: Z = 1.58 (P = .11)

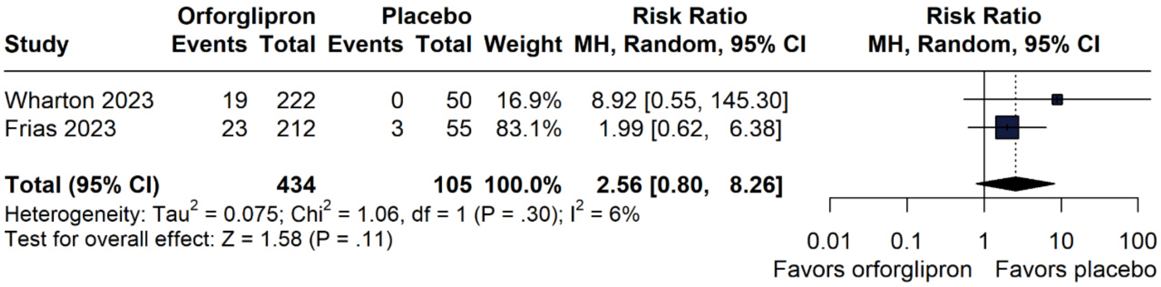

Supplementary Figure 6. Forest plots of pooled comparisons between orforglipron and placebo. (**A**) Diarrhea. (**B**) Dyspepsia. **(C**) Cardiac disorders.

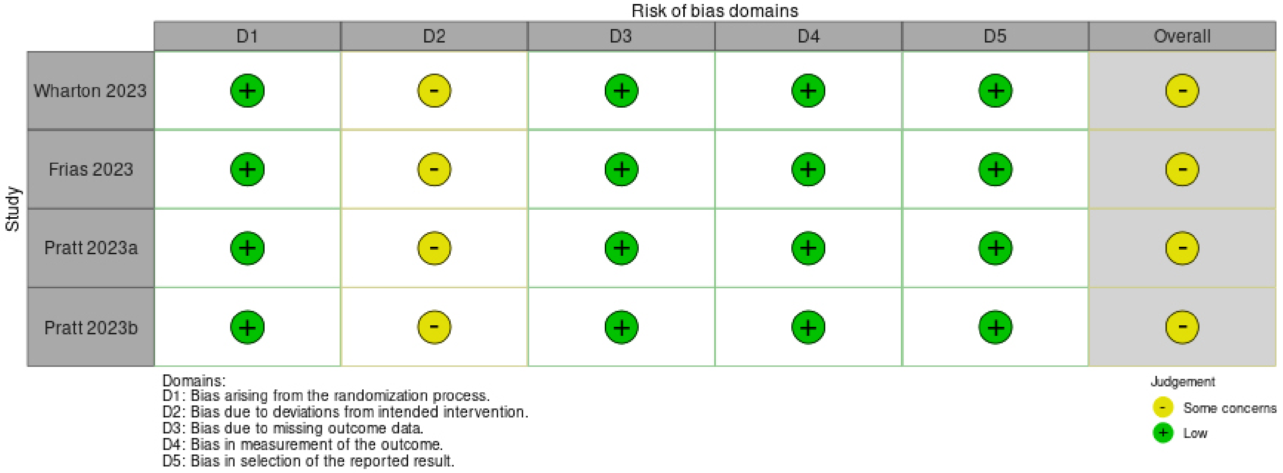

Supplementary Figure 7. Critical appraisal of randomized controlled trials according to the Cochrane Collaboration tool for assessing risk of bias in randomized trials.

Copyright© AE&M all rights reserved.

Supplementary Material 2

Effects of Once-Daily Oral Orforglipron on Weight and Metabolic Markers: A Systematic Review and Meta-analysis of Randomized Controlled Trials

Bibliography: Lütkemeyer et al.

Certainty assessment							Summary of findings				
Participants (studies) Follow-up	Risk of bias	Inconsistency	Indirectness	Imprecision	Publication bias	Overall certainty of evidence	Study event rates (%)		Relative effect (95% CI)	Anticipated absolute effects	
							With [Placebo]	With Orforglipron		Risk with [Placebo]	Risk difference with Orforglipron
**Weight Reduction (kg)**											
733 (4 RCTs)	serious[a]	serious[b,c]	not serious	not serious	none	⨁⨁◯◯ Low	137	596	-		MD **6.14 lower** (9.62 lower to 2.66 lower)
**BMI (kg/m²)**											
605 (2 RCTs)	serious[a]	serious[b,c]	not serious	serious[d]	none	⨁◯◯◯ Very low	105	500	-		MD **2.87 lower** (4.56 lower to 1.1 lower)
**Waist Circumference (cm)**											
605 (2 RCTs)	serious[a]	serious[b,c]	not serious	serious[d]	none	⨁◯◯◯ Very low	105	500	-		MD **5.32 lower** (9.13 lower to 1.51 lower)
**Weight Reduction ≥5%**											
438 (2 RCTs)	serious[a]	serious[b]	not serious	serious[d]	none	⨁◯◯◯ Very low	19/81 (23.5%)	276/357 (77.3%)	**RR 3.31** (2.23 to 4.93)	235 per 1,000	**542 more per 1,000** (from 289 more to 922 more)
**Weight Reduction ≥10%**											
438 (2 RCTs)	serious[a]	serious[b]	not serious	serious[d]	none	⨁◯◯◯ Very low	7/81 (8.6%)	168/357 (47.1%)	**RR 5.24** (2.07 to 13.31)	86 per 1,000	**366 more per 1,000** (from 92 more to 1,000 more)
**Weight Reduction ≥15%**											
438 (2 RCTs)	serious[a]	serious[b]	not serious	serious[d]	none	⨁◯◯◯ Very low	1/81 (1.2%)	80/357 (22.4%)	**RR 9.53** (1.26 to 71.89)	12 per 1,000	**105 more per 1,000** (from 3 more to 875 more)
**HbA1c (%)**											
673 (3 RCTs)	serious[a]	serious[b,c]	not serious	serious[e]	none	⨁◯◯◯ Very low	122	551	-		MD **0.84 lower** (1.54 lower to 0.14 lower)

CI: confidence interval; **MD:** mean difference; **RR:** risk ratio

Explanations

a. All studies had some concerns about risk of bias.
b. Heterogeneity between studies due to differences in baseline patient characteristics.
c. High heterogeneity.
d. Only 2 studies reported the outcome.
e. Only 3 studies reported the outcome.

This is an open-access article distributed under the terms of the Creative Commons Attribution License

Copyright© AE&M all rights reserved.
